# T cells drive negative feedback mechanisms in cancer associated fibroblasts, promoting expression of co-inhibitory ligands, CD73 and IL-27 in non-small cell lung cancer

**DOI:** 10.1080/2162402X.2021.1940675

**Published:** 2021-07-08

**Authors:** Richard A O’Connor, Vishwani Chauhan, Layla Mathieson, Helen Titmarsh, Lilian Koppensteiner, Irene Young, Guilia Tagliavini, David A Dorward, Sandrine Prost, Kevin Dhaliwal, William A Wallace, Ahsan R Akram

**Affiliations:** a Centre for Inflammation Research, Queen’s Medical Research Institute, University of Edinburgh, Edinburgh, UK; b Edinburgh Medical School, The Chancellor’s Building, University of Edinburgh, Edinburgh, UK; c Department of Pathology, Royal Infirmary of Edinburgh, Edinburgh, UK; d Department of Pathology, The Chancellor’s Building, University of Edinburgh, Edinburgh, UK; e Cancer Research UK Edinburgh Centre, Institute of Genetics and Cancer, The University of Edinburgh, Edinburgh, UK

**Keywords:** Non-small cell lung cancer, cancer-associated fibroblasts, T cells, crosstalk

## Abstract

The success of immune checkpoint therapy shows tumor-reactive T cells can eliminate cancer cells but are restrained by immunosuppression within the tumor micro-environment (TME). Cancer associated fibroblasts (CAFs) are the dominant stromal cell in the TME and co-localize with T cells in non-small cell lung cancer. We demonstrate the bidirectional nature of CAF/T cell interactions; T cells promote expression of co-inhibitory ligands, MHC molecules and CD73 on CAFs, increasing their production of IL-6 and eliciting production of IL-27. In turn CAFs upregulate co-inhibitory receptors on T cells including the ectonucleotidase CD39 promoting development of an exhausted but highly cytotoxic phenotype. Our results highlight the bidirectional interaction between T cells and CAFs in promoting components of the immunosuppressive CD39, CD73 adenosine pathway and demonstrate IL-27 production can be induced in CAF by activated T cells.

## Introduction

Stromal cells influence all phases of the immune response. From directing migration of naïve T cells in the lymph nodes and facilitating their proliferation to limiting clonal expansion and effector function, the appropriate activity of stromal cells optimizes immune responses.^1^ Cancer-associated fibroblasts (CAFs) however present an impediment to effective anti-tumor immunity,^2^ promoting tumor growth and immunosuppression.^3–5^ In non-small cell lung cancer (NSCLC) the majority of tumor-associated T cells are found within the stroma, amidst a dense network of CAFs, suggesting CAF/T cell interactions are likely to influence T cell function in situ. In the Lewis lung carcinoma model deletion of fibroblast activation protein (FAP)^+^ expressing CAFs slows tumor growth in an IFN-γ/TNF-α dependant fashion^6^ showing CAFs restrain anti-tumor immunity. Under inflammatory conditions fibroblasts can be induced to express MHC-II^7^ and uptake and process antigen^3^ increasing their potential for interactions with T cells. Expression of CD73 on CAFs limits T cell expansion via production of immunosuppressive adenosine.^8^ Thus CAF-mediated suppression of T cell function in the tumor microenvironment (TME) represents a critical late-stage barrier to effective anti-tumor immunity.

Tumor infiltrating T cells (TILs) in NSCLC display characteristics of T cell exhaustion including a hierarchical loss of effector function, expression of multiple co-inhibitory receptors and enhanced susceptibility to apoptosis.^9^ The most profound exhaustion is seen in tumor reactive T cells which, after multiple rounds of stimulation, are unable to effectively eliminate the target antigen.^10,11^ During chronic viral infection exhausted T cells express CD39 (NTPDase1)^12^ and single-cell RNA-sequencing identified an enrichment of exhausted CD39 expressing T cells in NSCLC tumor versus normal non-transformed lung tissue.^13^ Enrichment of CD39^+^ T cells is a common feature of solid organ malignancies including head and neck cancer, renal cell carcinoma and gastric adenocarcinoma.^14^ CD39 expression requires TCR stimulation^15^ and identifies T cells which have recently encountered antigen allowing differentiation of tumor-reactive CD39^+^ T cells from CD39^−^ bystander T cells.^10^ Consequently, there is particular interest in CD39 expression in TILs where expression allows enrichment for tumor-reactive T cells without knowledge of their fine specificity.^16^ CD39 is an ectonucleotidase which converts ATP/ADP to adenosine monophosphate decreasing the availability of pro-inflammatory ATP and promoting production of immunosuppressive adenosine.^17^ Loss of CD39 augments CD8^+^ T cell responses,^18^ inhibits its enzymatic activity increasing cytokine production^15^ and enhances anti-tumor responses.^19^ Although the suppressive effects of CD39 are best described in Tregs^17^ they have also been reported for CD39^+^ CD8^+^ T cells^20,21^ raising the possibility that CD39^+^ cytotoxic T cells (CTLs) may directly contribute to immunosuppression in the TME. The critical factors and cellular interactions driving CD39 expression in TILs are incompletely defined but modulating CD39 expression and/or function may provide an opportunity to slow development of the most profound T cell exhaustion and strengthen anti-tumor immunity.

A full understanding of factors in the TME promoting expression of multiple co-inhibitory receptors and their ligands, immunosuppressive pathways and pro and anti-inflammatory cytokines is necessary to direct the rational development of therapies. Many immunosuppressive elements of the TME, including upregulation of PD-L1, production of indolamine-2,3-dioxygenase (IDO) and elevated numbers of Tregs, are induced as a consequence of the anti-tumor immune response itself.^22^ In order to investigate how activated T cells influence the phenotype of CAFs and vice versa we measured phenotypic and functional changes in both populations during co-culture in primary NSCLC samples. Activated T cells increase expression of PD-1 ligands, MHC-II, CD73 and production of immunomodulatory cytokines (including IL-6 and IL-27) by CAFs, which in turn promoted expression of multiple co-inhibitory receptors including CD39, PD-1, Tim3 and production of IL-10 by T cells. The bidirectional interactions of CAF and T cells in the tumor stroma could act to promote immunosuppression and T cell exhaustion and provide a target to prevent loss of function in tumor-reactive T cells.

## Results

### Exhausted CD39^+^ T cells co-localize with FAP^hi^ CAFs in NSCLC

**Figure 1.**
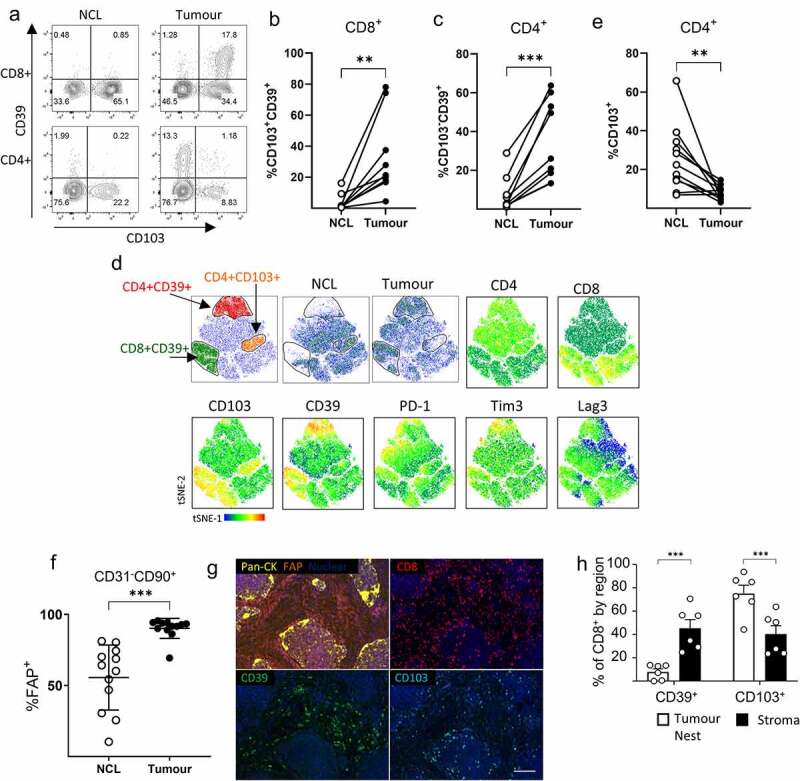
**CAF co-localize with C39^+^ T cells in the tumor microenvironment**. a) Representative staining of CD8^+^ (upper panels) and CD4^+^ (lower panels) T cells from paired non-cancerous lung (NCL) or tumor for CD39 and CD103. b) Proportion of CD8^+^ T cells co-expressing CD39 and CD103 in paired NCL/tumor samples (** *P* = .005). c) Proportion of CD103-CD39^+^ cells within CD4^+^ T cells from paired NCL and tumor samples (n = 9) (*** *P* = .0007). d) tSNE plots showing concatenated data files gated on CD45^+^CD3^+^ T cells from four paired NCL and tumor samples, gates indicate the location of CD8^+^CD39^+^ cells, CD4^+^ CD39^+^ cells and CD4^+^ CD103^+^ cells. The distribution of cells derived from non-cancerous lung (NCL) and tumor samples is shown and heat maps illustrate the relative expression of CD103, CD39, PD-1, Tim3 and LAG3. e) The frequency of CD4^+^ cells expressing CD103 in paired NCL and tumor samples (n = 11) (** *P* = .0075). f). FAP expression on CD45-EpCAM^−^CD31^−^CD90^+^ stromal cells derived from NCL or paired tumor samples (n = 12) (*** *P* = .0005). g). Representative Immunohistochemistry illustrating localistaion of FAP, CD8, CD39 and CD103 expressing cells in a section of NSCLC, scale bar represents 100 µm. h) Distribution of CD39^+^ and CD103^+^ CD8^+^ T cells illustrates enrichment of CD39^+^ cells within the stroma (*** *P* = .0008) and CD103^+^ cells within NSCLC tumors (*** *P* = .006) (n = 6). Two tailed paired T tests were used for all statistical analyses

In human NSCLC we found a locally elevated frequency of CD39^+^ T cells in the tumor versus paired non-cancerous lung (NCL) tissue (Figure 1a), consistent with published reports.^11,16^ This remains true in both CD4^+^ and CD8^+^ compartments (Figure 1(b,c)). Co-expression of CD39 and CD103 is common in CD8^+^ T cells but in stark contrast the majority of CD39^+^ CD4^+^ T cells are CD103^−^ (Figure 1a). Treg cells are enriched in the TME where they express high levels of CD39 (a feature of highly activated Tregs^23^ with enhanced stability of suppressive function at inflammatory sites^24^) and lack of expression of CD103 (Figure 1a Sup Figure 1a-c). Therefore, distinct patterns of CD39 and CD103 expression describe tumor-reactive CTLs (CD8^+^/CD39^+^/CD103^+^) and tumor infiltrating Tregs (CD4^+^/CD39^+^/CD103^−^). Combining data on CD3^+^ T cells from multiple paired tumor and non-cancerous adjacent lung tissues illustrates significant enrichment of both of these populations in cancerous tissue (Figure 1d). Among CD8^+^ CTLs, the CD39^+^ express the highest levels of multiple co-inhibitory molecules including PD-1, Tim3 and Lag3 (Figure 1d) as well as the hierarchical loss of effector function associated with the development of exhaustion. Reduced capacity for TNF-α production was most pronounced in CD39^hi^ CD103^+^ CD8^+^ T cells while these cells retained the highest capacity for granzyme B production and showed greater evidence of proliferation ex-vivo by Ki67 staining (Sup SFigure 2a), in line with the findings of Canale et al.^11^ in human breast cancer. CD39^+^ CD4^+^ T cells showed reduced TNF-αα production and lacked capacity for IFN-γ production as would be expected of Tregs. Notably we found a relative lack of CD4^+^CD103^+^ T cells in tumor versus non-cancerous adjacent lung tissue (Figure 1d/1 F) and that the small population of CD103^+^ cells remaining had the highest capacity for IFN-γ production (Supp Figure 2) illustrating a relative lack of CD4^+^ T cells with maximum effector function in the TME.

**Figure 2.**
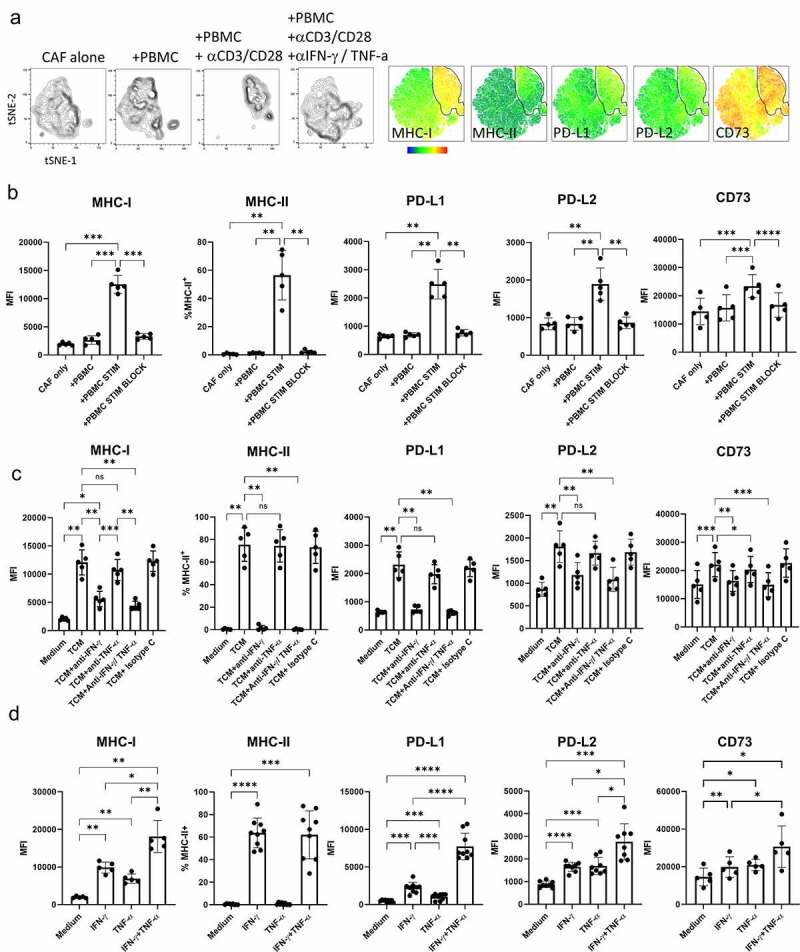
**IFN-γ and TNF-α produced by activated T cells upregulated expression of MHC, PD-1 ligands and CD73 on CAF**. Five independently generated NSCLC CAF lines were cultured alone (CAF-only), or in the presence of PBMC (+PBMC), PBMC + anti-CD3/anti-CD28 (PBMC +STIM) or PBMC + anti-CD3/anti-CD28 with the addition of neutralizing antibodies to IFN-γ and and-TNF-α (PBMC+STIM+BLOCK) for 48 hrs. fcs files gated on CD90^+^ CAFs were concatenated and used to generate tSNE plots. **a)** tSNE plots showing the distribution of CAFs cultured under each condition, heat-maps show expression levels of MHC-I, MHC-II, PD-L1, PD-L2 and CD73. The gate shows the position of CAFs cultured in the presence of activated T cells. **b)** Expression levels of MHC-I, PD-L1, PD-L-2 and CD73 are shown as MFI and the expression of MHC-II (% +) on CAFs cultured under the conditions described above. **c)** Five CAF lines were cultured alone (Medium) or in the presence of supernatants derived from CD3 stimulated tumor infiltrating T cells (TCM) with the addition of neutralizing antibodies to either IFN-γ or TNF-α, to both IFN-g and TNF-α or of appropriate isotype matched control antibody as indicated. d) CAF lines were cultured alone of in the presence of either rIFN-γ or rTNF-α (both used at 25 ng/ml) or a combination of IFN-γ and TNF-α n = 5 to 8 independently generated CAF lines. Data are representative of three experiments with 2 to 5 CAF lines per experiment. One way ANOVA was used for all statistical analysis with Tukey’s multiple comparisons posttest (ns = not significant,* *P* = <0.05, ** *P* = <0.01, *** *P* = <0.001, ****P = <0.0001)

CAFs in NSCLC can be differentiated from normal fibroblasts in non-cancerous adjacent lung tissue by elevated expression of fibroblast activation protein (FAP) (figure 1f, gating strategy Sup Figure 3). High levels of FAP expression in the stromal compartment of the tumor is associated with T cell retention in the stroma (Sup SFigure 4) where CD39^+^ CD8^+^ T cells are found in close association with FAP^+^ CAFs (Figure 1(g, h)/Sup SFigure 4).

**Figure 3.**
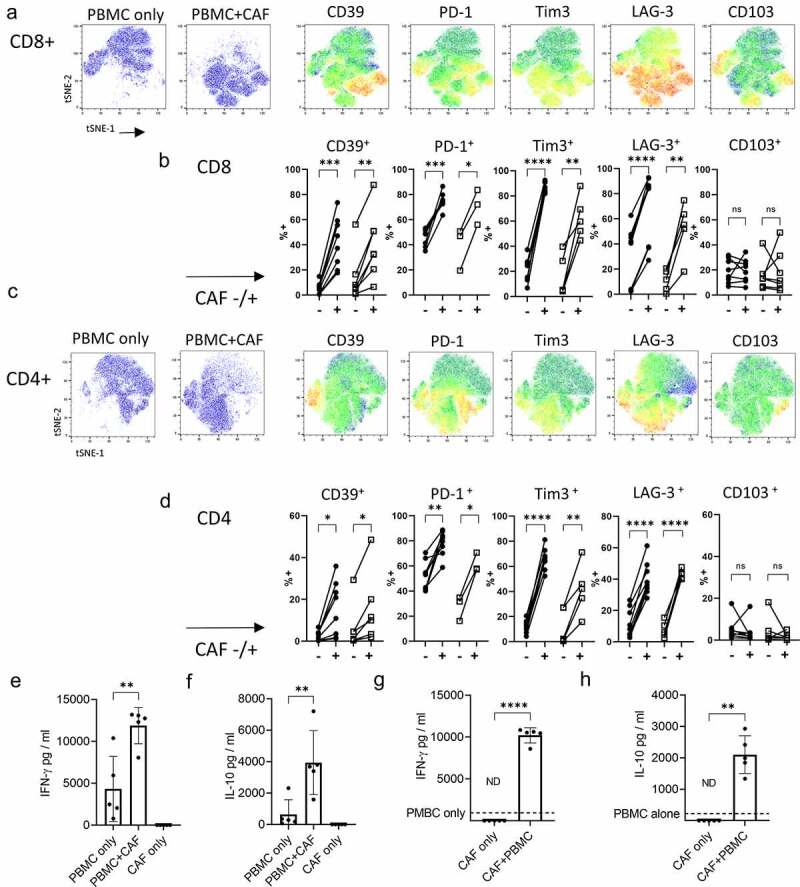
**CAF promote expression of co-inhibitory molecules and alter cytokine production by activated T cells**. PBMC from five healthy donors were stimulated with anti-CD3/anti-CD28 antibodies either alone (PBMC-only) or in the presence of CAF (PBMC+CAF) after 48 hrs stimulation cells were recovered and stained for analysis by flow cytometry. Concatenated fcs files gated on CD8^+^ (a) or CD4^+^ (c) T cells showing distribution in culture with PBMC only or in the presence of CAF. Heat-maps show the relative level of expression of CD39, PD-1, Tim3, LAG-3 and CD103. Expression of CD39, PD-1, Tim-3, LAG-3 and CD103 on CD8^+^ (b) and CD4^+^ (d) T cells from healthy donors (n = 8 closed circles) or NSCLC patients (n = 3–6 open squares). **e)** IFN-γ production by PBMC stimulated in the presence or absence of CAFs. (f) IL-10 production by PBMC stimulated in the presence or absence of CAFs. Production of (g) IFN-γ and (h) IL-10 by 5 independently generated NSCLC CAF lines were cultured alone or in the presence of PBMC activated with anti-CD3/CD28 antibodies. Error bars show standard deviation. Two-tailed paired T tests were used for all statistical analyses (ns = not significant,* *P* = <0.05, ** *P* = <0.01, *** *P* = <0.001, ****P = <0.0001)

**Figure 4.**
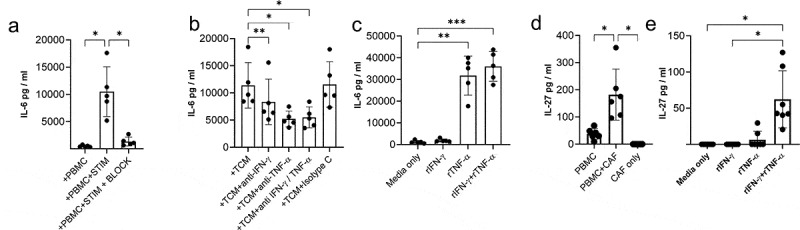
**Cytokines produced by activated T cells promote production of IL-6 and IL-27 by CAF**. IL-6 production a) by five independently generated NSCLC CAF lines co-cultured with either PBMCs only (+PBMC), with PBMCs +anti-CD3/anti-CD28 antibodies (+PBMC+STIM) or with PBMC+anti-CD3/anti-CD28 antibodies and neutralizing cytokines to IFN-γ and TNF-α. b) IL-6 production by CAF cultured in the presence of conditioned media from anti-CD3 stimulated tumor infiltrating T cells (TCM) pre-incubated with neutralizing antibodies to either IFN-γ, TNF-α or both IFN-γ/TNF-α. c) IL-6 production by CAF in response to rIFN-γ, rTNF-α (each at 25 ng/ml) or a combination of rIFN-γ and rTNF-α. d) IL-27 production in cultures of PBMC stimulated with anti-CD3/anti-CD28 alone or when in co-culture with CAF. E) IL-27 production by seven independently generated NSCLC CAF lines stimulated with rIFN-γ or rTNF-α (both used at 25 ng/ml) or a combination of IFN-γ and TNF-α. Results are pooled from three experiments. One-way ANOVA was used for all statistical analysis with Tukey’s multiple comparisons posttest (* *P* = <0.05, ** *P* = <0.01, *** *P* = <0.001, ****P = <0.0001)

### T cell activation promotes expression of co-inhibitory ligands, MHC molecules and CD73 on CAF

Co-culture of five independently generated NSCLC CAF lines with PBMC revealed a distinct pattern of responsiveness to activated T cells. T cell activation resulted in increased expression of MHC-I, induction of MHC-II expression, elevated expression of the co-inhibitory ligands PD-L1 and PD-L2 and increased expression of the ectonucleotidase CD73 (Figure 2a,b). Anti-CD3/CD28 stimulated PBMC produced around 4322 pg/ml IFN-γ and 75 pg/ml TNF-αα and changes in co-inhibitory molecule expression could be reversed by neutralization of IFN-γ and TNF-αα (Figure 2a, b). Increased co-inhibitory ligand expression could also be induced by culture of CAFs in the presence of conditioned media from activated T cells ((Figure 2c) which contained 3224 pg /ml IFN-γ and 547 pg/ml TNF-α). The effect of IFN-γ was dominant in modulating MHC-I, PD-L1/2 and CD73 expression and was solely responsible for induction of MHC-II expression (Figure 2c). However, clear synergistic effects of co-exposure to IFN-γ and TNF-αα resulted in the highest levels of MHC-1, PD-L1 and PD-L2 (Figure 2d). Thus, activated T cells induce phenotypic changes in CAFs which may both increase the capacity of CAFs for antigen presentation and suppress further T cell activation via PD-1 interactions with PD-L1/PD-L2 or adenosine production by CD73.

**CAFs promote expression of multiple co-inhibitory receptors including CD39 and increase production of IFN-**γ **and IL-10 in activated T cells**

Activation in the presence of CAFs induced phenotypic changes in both CD8^+^ (Figure 3a,b) and CD4^+^ (Figure 3c, d) T cells with upregulation of co-inhibitory receptors PD-1, Tim3 and LAG-3 as well as CD39 (Figure 3a,b) resulting in a phenotype resembling that of tumor infiltrating T cells (Figure 1a). Significant upregulation of CD39, PD-1, Tim3 and LAG-3 was seen in multiple experiments but CD103 levels were not enhanced by CAFs (Figure 3a-d). Pre-exposure of CAF to IFN-γ and TNF-α (to increase PD-L1 and PD-L2 expression) prior to co-culture with PBMCs did not further increase the degree of CAF-induced co-inhibitory molecule expression suggesting increased PD-1-signaling does not account for the increased co-inhibitory receptor expression (Sup SFigure 4). Co-culture also increased production of IFN-γ and IL-10 (Figure 3e, f). CAF did not increase the frequency of IFN-γ producing cells but rather increased the amount of cytokine produced by both CD8^+^ and CD4^+^ T cells (Figure 3e and Sup SFig 6). CAF significantly increased both the amount of IL-10 produced (Figure 3e) and the frequency of IL-10 producers among CD8^+^ but not CD4^+^ T cells (Sup SFigure 5). CAFs themselves did not produce either IFN-γ or IL-10 (Figure 3e, f). The cytokine enhancing function of CAF is consistently seen as five independently generated NSCLC CAF lines showed capacity to upregulate IFN-γ (Figure 3g) and IL-10 (Figure 3h) production when in co-culture with activated T cells.

### Activated T cells elevate production of IL-6 and induce production of IL-27 in CAFs

Having shown CAFs actively promote the development of key phenotypic characteristics of exhausted tumor infiltrating lymphocytes and production of the immunosuppressive cytokine IL-10 we sought to identify factors produced by CAFs which might drive these changes. Induction of CD39 requires TCR stimulation (in vitro and in vivo^11,15,25^) and IL-6,^11^ IL-27^26^ and TGF-β^16,27^ have been reported to increase CD39 expression. The ability of CAF to produce IL-6 is well documented and can have pro-tumor effects.^28^ Activated T cells significantly increased IL-6 production by CAFs and this was blocked by neutralizing TNF-α and IFN-γ (Figure 4a). T cell-conditioned media (containing 3224 pg /ml IFN-γ and 547 pg/ml TNF-α) also increased IL-6 production by CAFs and this was inhibited by neutralizing TNF-α and to a lesser extent IFN-γ (Figure 4b). Confirmatory experiments with recombinant cytokines showed TNF-α is most potent in driving IL-6 by NSCLC CAFs (Figure 4c).

IL-27 favors development of Th1 responses during T cell priming but also exerts potent effects on activated T cells including promoting production of IL-10^29,30^ and inducing expression of multiple co-inhibitory receptors.^31^ While none of the nine NSCLC derived CAF lines tested produced IL-27 spontaneously we were surprised to discover that activated T cells (which produced around 4322 pg/ml IFN-γ and 75 pg/ml TNF-α) induce production of IL-27 by fibroblasts (Figure 4d). IL-27 was induced in CAFs by the synergistic action of recombinant TNF-α and IFN-γ confirming the capacity of CAF to produce IL-27 in the absence of other cell types (Figure 4e). Thus IFN-γ and TNF-α act in concert to upregulate components of the antigen presentation pathway, maximize expression of PD-1 ligands, CD73 and production of IL-6 and IL-27 by CAF.

### CAF induce CD39 expression in activated T cells via TGF-βsignaling

**Figure 5.**
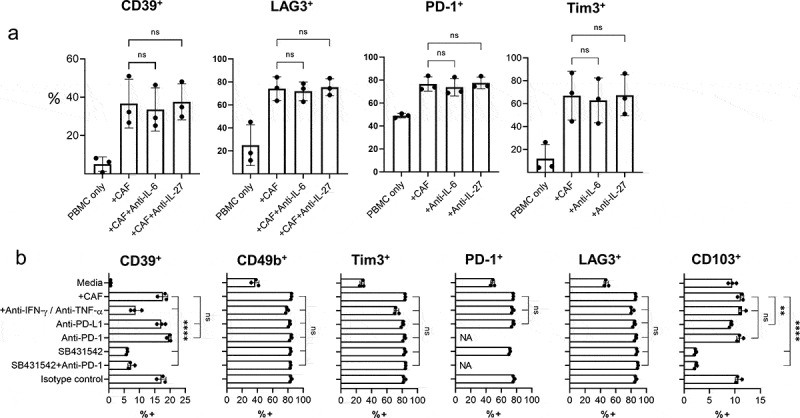
**CAF-induced CD39 expression can be inhibited by neutralizing IFN-γ and TNF-**α **or by inhibition of TGF-β signaling a)** Expression of CD39, LAG-3, PD-1 and Tim3 on CD8 + T cells activated either alone (PBMC-only) or in co-culture with CAF (+CAF) in the presence of neutralizing antibody together IL-6 or IL-27. **b)** Expression of CD39, CD49b, Tim3, PD-1 LAG3 and CD103 on activated CD8^+^ T cells either cultured alone (media) or in co-culture with CAF (+CAF) and neutralizing antibodies to IFN-γ and TNF-α, anti-PD-L1, anti-PD-1, SB431542, SB431542+ anti-PD-1 or isotype control antibody as indicated. Error bars show standard deviation of triplicate wells. Significance shown for each parameter when compared to +CAF. Results for SB431542 are representative of those from three repeat experiments using different PBMCs. For PD-1, NA where PD-1 blockage used with PD-1 surface levels. One way ANOVA was used for all statistical analysis with Tukey’s multiple comparisons posttest (ns = not significant,* *P* = <0.05, ** *P* = <0.01, *** *P* = <0.001, ****P = <0.0001)

Although IL-6 and IL-27^11^ have been shown to promote CD39 expression during T cell activation, neutralizing antibodies to IL-6 or IL-27 did not inhibit CAF-induced CD39 expression in activated CD8^+^ T cells (Figure 5a and Sup Fig 7). Neither did recombinant IL-27 increase CD39 expression in T cells during stimulation of PBMCs in the presence or absence of CAF (Sup SFig 7) or increase expression of CD39 or PD1 ligands in CAF (Sup SFig 8). TGF-β can also promote CD39 expression^16^ and addition of SB431542 (a selective inhibitor of TGF-β signaling) significantly reduced levels of CAF-induced CD39 expression (Figure 5b). SB431542 also reduced levels of CD103 expression demonstrating its effective suppression of TGF-β signaling (Figure 5b). Enhanced PD-1 signaling during co-culture with CAFs, which express high levels of PD-L1/PD-L2, did not promote CD39 expression as blockade of PD-1 signaling with either anti-PD-1 or anti-PD-L1 antibodies did not reduce CD39 expression. Neither did PD-1 blockade enhance suppression of CD39 expression by TGF-β inhibition, illustrating TGF-β mediated upregulation of CD39 occurs independently of PD-1 signaling (Figure 5b). Interestingly neutralizing IFN-γ and TNF-α also suppressed CAF-induced CD39 expression indicating cytokine production by T cells during activation potentiates the capacity of CAFs to induce CD39 expression (Figure 5b). CD39 expression was modulated independently of other exhaustion associated co-inhibitory receptors PD-1, Tim3, or LAG-3 (Figure 5b) which were not influenced by IL-6, IL-27, IFN-γ/TNF-α, TGF-β or PD-1.

Our results describe a scenario wherein IFN-γ and TNF-αβ produced by activated T cells promote upregulation of MHC molecules, co-inhibitory ligands and the immunosuppressive ectonucleotidase CD73 on the surface of CAFs as well as elevating their production of IL-6 and inducing production of IL-27. In turn CAFs induce characteristics of T cell exhaustion including upregulation of multiple co-inhibitory molecules such as PD-1, Tim3 and LAG-3 and the TGF-β mediated upregulation of CD39 replicating the phenotypic characteristics of the most highly activated tumor reactive cells in CAF-rich stroma of NSCLC tumors.

## Discussion

Activated fibroblasts have the potential to uptake, process and present antigen to T cells^32^ and the consequences of CAF/T cell interactions in the tumor microenvironment can dictate the efficacy of anti-tumor immune responses.^3,6,8,33,34^ Although low co-stimulatory molecule expression means fibroblasts are generally inefficient compared to professional antigen presenting cells they can activate memory T cells^35^ and in the right cytokine environment can initiate immune responses.^32^ Single cell RNA sequencing has recently identified MHC-II expressing CAF with the capacity to present to CD4^+^ T cells in pancreatic ductal adenocarcinoma highlighting the potential for CAF to interact with T cells in the TME.^36^ The majority of tumor infiltrating T cells are previously activated, displaying features of a tissue resident memory (TRM) phenotype^37^ and co-expressing multiple co-inhibitory molecules.^9,10,13^ Though these cells show signs of exhaustion they are not entirely dysfunctional^38,39^ but rather exquisitely sensitive to environmental conditions. The success of immune checkpoint therapy shows that reducing the negative signals received by tumor reactive T cells can be sufficient to unleash effective anti-tumor immunity. While targeting individual signaling pathways such as PD-1/PD-L1 or CTLA-4 can be effective it is the overall balance of positive and negative signals which determines outcome and justifies the many combined approaches to immunotherapy currently under study.

The beneficial effects of targeting CAFs, in experimental models, rely on the presence of an adaptive immune response^6,40^ and increase the effectiveness of checkpoint inhibitors indicating CAFs suppress T cell responses and facilitate suppression via PD-1 in vivo.^33^ In vitro studies show CAFs can limit T cell proliferation,^41^ alter patterns of cytokine production,^42–44^ promote apoptosis^3^ and upregulate co-inhibitory receptor expression.^44^ We have extended these findings to show human NSCLC-derived CAFs also induced expression of CD39 on activated T cells via TGF-β. There is increasing interest in CD39^+^ T cells in the TME due to the demonstration that CD39 expression identifies tumor reactive CTLs.^10,16^ The frequency of CD39^+^ cells in lung cancer also correlates with the mutation status of the epidermal growth factor receptors on tumor cells suggesting high levels of neoantigen presentation are reflected in a higher frequency of exhausted responder T cells.^10^ Re-invigorating exhausted tumor reactive T cells is the goal of immunotherapy and CD39^+^ T cells display an exhausted phenotype expressing the highest levels of multiple co-inhibitory receptors including PD-1/Tim3 and LAG-3.^11,16^ Cells expressing multiple co-inhibitory receptors are the most profoundly functionally impaired and likely to require inhibition of multiple immune checkpoints to regain effective function.^45^ PD-1^hi^ tumor infiltrating T cells typically express multiple co-inhibitory receptors and their frequency is predictive of both responsiveness to PD-1 blocked and survival in NSCLC patients^38^ suggesting reinvigoration is possible and that the presence of PD-1^hi^/CD39^+^ T cells in the TME indicates the potential exists for an effective anti-tumor immune response. Our results suggest that T cell activation in the TME modulates CAF function igniting a negative feedback mechanism which raises PD-1 expression along with CD49b, Tim3, LAG-3 and CD39 on activated T cells whilst simultaneously elevating PD-L1/PD-L2 and CD73 expression on CAFs themselves.

IFN-γ displays a dual role in tumor immunology, it promotes immunity via activation of cytotoxic T cells and upregulating expression of MHC-I molecules on tumor cells, but also promotes suppression by inducing expression of PD-1 ligands (Reviewed)^.[46]^ Similarly, while IFN-γ upregulates expression of MHC-I and induces expression of MHC-II in fibroblasts^7^ it promotes expression of PD-L1 and PD-L2^42^ and CD73. PD-1/PD-L1 interactions control the magnitude of immune responses and contribute to peripheral tolerance and dynamic regulation of PDL-1 expression allows efficient local regulation. The highest levels of PD-L1 expression are found in the most immunologically active tumors demonstrating links between the vigor of the anti-tumor response and the suppressive nature of the TME.^47^ While PD-L1 can be induced in many cell types expression of PD-L2 is mostly restricted to antigen presenting cells. However, CAFs express higher levels of PD-L2 than normal fibroblasts in lung, colon, pancreatic and breast cancer^3^ and PD-L2 mediated signaling has been implicated in CAF mediated immunosuppression.^3^ In breast cancer distinct CAF subtypes have been described and the dominant FAPhi subset of immunosuppressive CAF express the highest levels of PD-L2 and CD73.^4^ IFN-γ and TNF-α show synergistic activity in regulating many aspects of immunity including production of iNOS^48^ IL-6,^49^ IL-8 and CXCL-10.^50^ IFN-γ and TNF-αα also promote the highest level of costimulatory molecule expression in fibroblasts.^51^ In addition, we found they have synergy in eliciting the highest observed level of PD-L1/PD-L2 and CD73 expression in NSCLC CAFs as well as increasing production of IL-6 and initiating production of IL-27. Besides IFN-γ and TNF-α IL-6 and IL-27 can also promote PD-L1 expression^52^ and their production may further amplify upregulations induced by IFN-γ and TNF-α. The finding that IFN-γ and TNF-α induce IL-27 production by CAFs identifies a novel immunoregulatory function in CAF ignited by activated T cells. IL-27 is a heterodimeric cytokine of the IL-6/IL-12 cytokine families composed of the Epstein–Barr virus (EBV) induced gene 3 (EBI3) and the IL-27p28 subunits and plays important roles in the initiation and regulation of immune responses (reviewed)^.[53]^ IL-27 has potent anti-tumor effects both directly,^54^ via inhibiting angiogenesis^55^ and in promoting granzyme B expression and expansion of effective anti-tumor T cells.^56–58^ However IL-27 can also restrain immune responses, it promotes CD39 expression in tumor infiltrating Tregs,^26^ drives expansion of IL-10 producing Tr1 cells^30,59^ and increases expression of multiple co-inhibitory receptors^31^ as well as the co-inhibitory ligands PD-L1 and PD-L2.^60^ IL-27 promotes expression of the co-inhibitory receptor Tim3 and production of IL-10^61^ and in the absence of IL-27 R-signaling tumor infiltrating T cells retain functionality and more effectively prevent tumor growth, confirming its relevance to T cell responses in the TME. While we were unable to demonstrate a role for IL-27 in CAF-mediated induction of CD39, Tim3 or IL-10 in T cells it is possible that IL-27 signaling was not entirely blocked by our neutralizing antibody to IL-27. Huang et al^62^ recently described of a role for IL-27 in maintaining the capacity of CD8^+^ T cells to proliferate and avoid apoptosis during chronic inflammation by promoting IRF1 expression. This highlights the potential of IL-27 to support the maintenance of a pool of T cells ready to respond to checkpoint therapy and raises the possibility that activated stromal cells could positively impact T cell survival via IL-27 production.

Fibroblasts express both chains of the IL-27-receptor, WSX-1 and Gp130, and respond to IL-27 with STAT-1 activation and increased expression of IL-6, CXCL10 and ICAM-1.^63,64^ While several studies have shown responsiveness of fibroblasts to IL-27, evidence of IL-27 production by fibroblasts has only been reported in response to the synthetic TLR3 ligand poly I:C.^65^ Collectively these studies indicate fibroblast responses to IL-27 have the potential to impact both tumor cell growth and T cell responses. Whether IL-27 production by CAF occurs in the TME and exerts autocrine effects on fibroblasts and/or paracrine effects on T cells in the TME will be the source of future investigation.

CD39 expression can shape immunity in the long term, interfering with the formation T follicular helper cells^66^ and long lived memory T cells.^67^ Expression of CD39 on CD4^+^ T cells in aged individuals correlates with increased susceptibility to apoptosis which can be reversed by CD39 inhibition illustrating a role for CD39 in regulating T cell longevity.^67^ Increased susceptibility to apoptosis is also a feature of tumor infiltrating T cells and limits effective anti-tumor immunity^47^ and increasing CD39 expression may represent a novel means by which CAF favor T cell apoptosis in the TME. CD39 expression also limits CD8^+^ T cell responses in atherosclerotic lesions,^15^ during chronic viral^12^ and bacterial infection^18^ providing a common suppressive pathway in chronic inflammation. Our results suggest stromal cells, by promoting CD39 expression in T cells, can contribute to the generation of a locally suppressive microenviroment. During chronic inflammation this may represent a means of retaining responsiveness to the initiating antigen while preventing activation or limiting expansion of newly recruited T cells, potentially responsive to autoantigens released during inflammation, and thus preventing epitope spreading. While this would be desirable in true autoimmunity it would limit responsiveness to neoantigens and hinder effective immunity in cancer.

Functionally CD39 can act in concert with CD73 to promote immunosuppression by converting ATP to immunosuppressive adenosine.^17^ Adenosine contributes to immunosuppression within the TME^68^ and the CD39 antagonist ARL-67156 enhances the function of intratumoral T cells.^69^ Deletion of the A2A adenosine receptor increases the efficiency of anti-tumor CAR T cells.^70^ Mixed bone marrow chimera experiments show that CD73 expression in CAFs limits T cell response and prevents tumor rejection.^8^ Consequently there is increasing interest in targeting these ectoenzymes as inhibitory checkpoints in cancer. While Tregs express both ectonucleotidases^17^ tumor infiltrating CTLs in NSCLC do not express CD73^11^ and while some studies report CD39^+^ CTLs can suppress the proliferation of CD39- responder cells^21,25^ others found they lacked suppressive potential.^11^ Notably CD39 and CD73 can synergistically promote immunosuppression even when expressed on different cell types as has been demonstrated in the case of suppressive Foxp3- Tr1 cells.^71^ High level expression of CD73 is a feature of the most immunosuppressive CAF subset in breast cancer^4^ and colorectal cancer^8^ and we found activated T cells further increased CD73 expression in NSCLC CAFs.

The ability of cancer associated fibroblasts to modify their phenotype in response to anti-tumor immunity presents a dynamic barrier to effective immunotherapy, calibrating the suppressive capacity of CAFs to the magnitude of the immune response. In addition to the classically described regulation via PD-1 we have shown that CAF/T cell interactions upregulate the components of adenosine mediated immunosuppression increasing expression of CD73 on CAF and CD39 of T cells as well as driving IL-27-production. The burgeoning interest in combined targeting of PD-1-signaling and adenosine production should factor in the potential role of CAFs in amplifying these pathways on the frontline of anti-tumor immunity.

### Materials and Methods

#### Ethics Statement

Healthy volunteer blood was obtained following informed consent and the study was approved by Lothian Regional Ethics Committee (REC) (REC No: 20-HV-069) prior to enrollment in the studies.

#### Samples and NSCLC tissue digestion

Cancer/lung tissue and blood samples were obtained following approval by NHS Lothian REC and facilitated by NHS Lothian SAHSC Bioresource (REC No: 15/ES/0094). All participants provided written informed consent NSCLC tissues and adjacent non-cancerous lung samples were collected from patients undergoing surgical resection with curative intent. Tumors >30 mm in diameter had areas from within macroscopic tumor and distal non-cancerous lung dissected by the attending pathologist. Fresh samples were processed immediately or stored in media overnight, then minced as finely as possible with scissors in bijoux prior to incubation with 1 mg/ml Collagenase IV (Merck), 1 mg/ ml DNase 1 (Merck), 50 U/ ml hyaluronidase (Stemcell Technologies) in DMEM (Life technologies) for 1 hr at 37°C with agitation. After digestion, samples were passed through 100 µM filters and then 70 µM filters to remove debris and centrifuged at 350 x g for 5 mins at room temperature. Supernatant was removed prior to red cell lysis (Merck) and counting. Typically single cell suspensions were surface stained and analyzed directly by flow cytometry. Cells for cryopreservation were stored in recovery media®(Thermo Fisher) and frozen at −80^o^C in overnight before transfer to liquid nitrogen storage.

PBMC from NSCLC patients were isolated from EDTA anti-coagulated whole blood samples collected 1 day prior to surgery and isolated using lymphoprep (Stemcell technologies). PBMC from healthy donors were obtained from consented adults in accordance with local regulations. When not used immediately PBMC were cryopreserved in liquid nitrogen.

#### Generation of T cell conditioned media

Sections of tumor samples around 1 cm^3^ were embedded in low melting point agarose and 400 µM slices cut using a Compresstome® VF-300-0Z vibrating microtome (precisionary). Individual slices were cultured in 24 well plates with media containing 3000 IU IL-2 (Gibco) until cells grew out to cover the bottom of the well (around 1 week). Cells were maintained in IL-2 containing media at a density of 0.8–2 x 10^6^/ml. T cells (>98% CD3+) were plated in 12 well plates at 5 x 10^5^/ml and stimulated with anti-CD3/anti-CD28 (both 1 µg/ml Bio-Xcell/Biolegend) for 48 hrs. After 48 hours cells were harvested centrifuged at 350 x g for 5 minutes and supernatants were drawn off, filtered through 0.22 um syringe filters and stored at −20 until use. Conditioned media were added to CAF cultures at a 1:1 vol:vol ratio (unsupplemented CAF cultures had half the media replaced at the same time to control for dilution effects).

#### Generation of CAF lines

Single cell suspensions from NSCLC tumors were incubated overnight in DMEM 100 U/ L penicillin/streptomycin, 2 mM L-glutamine and 10% FCS (all Gibco). Non-adherent cells were washed away and remaining cells grown to confluence in media supplemented with 1 X Insulin-Transferrin-Selenium. At passage cells were washed in PBS prior to treatment with 0.5% Trypsin EDTA for three minutes at 37°C to lift cells. At the third passage (when uniform CAF lines were free of non-CD90+ cells) CAF lines were cryopreserved. All CAF lines used were at passage 3–6.

#### CAF culture/co-culture conditions

For phenotypic analysis CAFs were plated at 4 x 10^4^/well in six well plates (Corning) and allowed to adhere and establish prior replacing media with or without addition of 10 ng/ ml rIFN-γ (Biolegend), 25 ng/ml rTNF-α (Biolegend), rIL-27 (Biolegend, as indicated) or a combnation of rIFN-γ and rTNF-α as indicated. For co-culture with PBMCs CAF were plated at 1 × 104 well in 1 ml in 24 plates (Corning) and cultured overnight to allow adherence prior to addition PBMC (5 x 105/well in 1 ml). At the time PBMCs were added to initiate co-cultures CAF only wells had 1 ml of media added at the same time to control for dilution effects. PBMC were pre-incubated with neutralizing antibodies for 30 minutes prior to adding them to CAF cultures as indicated. The following antibodies were used in cell culture anti-IFN-γ (clone B27 10 µl/ml) anti-TNF-α (clone Mab1 10 µg/ml) anti- IL-6 (clone MG2-13A55 10 µg/ml) all Biolegend. Anti-IL-27 (AF2526 2 µg /ml RnD systems). Anti-PD-1 (clone J116), anti-PD-L1 (clone 29E-2A3) mouse IgG1 (clone MOPC-21) mouse IgG2b (clone MPC-11) all Bio-Xcell. To inhibit TGF-β, the ALK inhibitor SB431542 was used at 10 µM (TOCRIS). After 48 hrs supernatants were harvested, centrifuged at 350 x g, sterile filtered and stored at −20°C for cytokine analysis and cells were harvested and stained for analysis by flow cytometry.

#### ELISAs

Performed according to manufacturers’ instructions for IL-10, IL-6 and IFN-γ (Biolegend) and IL-27 (RnD systems).

#### Flow cytometry

Cells were washed in PBS, dead cells stained with Zombie UV (Biolegend) according to the manufacturers instructions. Fc receptors were blocked with Trustain FcX (Biolegend) prior to staining with the indicated monoclonal antibodies in PBS 2% FCS (see details in supplementary methods). After staining cells were fixed in 2% PFA (Biolegend). Intranuclear staining (for Foxp3/Ki67) was performed after permeabilization with Foxp-permeabilization buffer (Invitrogen). Intracellular cytokine staining was performed using BD cytofix/cytoperm buffers according to the manufacturers instructions. Prior to staining for intracellular cytokines cells were incubated for 4hrs with 1X Cell activation cocktail (containing Phorbol-12-myristate 13-acetate, ionomycin, brefeldin A and monensin (invitrogen)) to stimulate cytokine production. All FACS data was collected on a 6 laser LSR (BD) and analyzed using Flowjo Software (BD). Populations of interest were downsampled to 4000 events and files were concatenated for tSNE analysis, ex-vivo tumor versus non-cancerous lung or in vitro treatment groups were identified using the sample ID parameter.

#### IHC/IFF

Formalin fixed paraffin-embedded slides of NSCLC resections (n = 22; 11 Adenocarcinoma, 8 squamous cell carcinoma), and one each of adenosquamous, large cell neuroendocrine and atypical carcinoid) were deparaffinised, rehydrated and antigen retrieval was undertaken with citrate buffer (Abcam ab64214) for 3 × 5 minutes in a microwave. Slides were processed with a commercial DAB staining kit (R&D Sytems, CTS019). Primary antibodies included FAP or CD3 incubated overnight at 4°C on sequentially cut slides. Secondary antibodies were added at RT for 1h and DAB was developed. Slides were counterstained with hematoxylin and mounted. Slides were scored for stromal FAP intensity or stromal CD3 presence on a 4-point scale; 0-no staining, 1-low staining, 2-moderate staining, 3-high staining.

For IFF, six slides with high FAP expression demonstrated by IHC had antigen retrieval undertaken with 0.125 mM EDTA buffer at 110°C for 30 minutes. Primary antibody concentrations were optimized to avoid cross-binding between the steps. Slides were incubated with Pan-CK at RT for 30 minutes followed by development using an AF488 Tyramide Superboost kit (Thermofisher, B40912). Microwave antigen retrieval with EDTA buffer was carried out before samples were sequentially incubated with primary antibody, species appropriate secondary, Qdot conjugation (followed by FAB blocking antibody where appropriate) and blocked for avidin & biotin (Vector Laboratories, SP-2001) and serum-free protein block (Dako, X0909) prior to next primary antibody. The following primary and Qdot conjugates were used; CD39 and Qdot 605, CD8 and Qdot 585, FAP and Qdot 565, CD103 and Qdot 705. Samples were then incubated with Qred for nuclear staining followed by rehydration and mounting. Slides were imaged on Vectra Polaris Imaging System (Akoya Biosciences). Imaging cubes were analyzed using inform software (Akoya Biosciences, v 2.1.10) with masks created for tumor and stromal areas, and the number of cells per area quantified.

#### Statistical analysis

One-way ANOVA with Tukey’s multiple comparisons posttest was used for comparison of multiple groups (* *P* = <0.05, ** *P* = <0.01, *** *P* = <0.001, ****P = <0.0001). Paired T tests were used for comparison of two experimental groups. All statistical analysis was performed using Graphpad Prism software.

## Reagent table and RRID identifiers

**Table:** 

REAGENT or RESOURCE	SOURCE	IDENTIFIER
Antibodies		
Ultra-LEAF™ Purified anti-human TNF-α Antibody	Biolegend	Biolegend Cat#502815 Clone MAb1 RRID:AB_2814397 (BioLegend Cat. No. 502805)
Ultra-LEAF™ Purified anti-human IFN-γ Antibody	Biolegend	Biolegend Cat#506532 Clone B27 RRID:AB_2801092 (BioLegend Cat. No. 506532)
Ultra-LEAF™ Purified anti-human IL-6 Antibody	Biolegend	Biolegend Cat# 501126 Clone MQ2-1385 RRID:AB_2810626 (BioLegend Cat. No. 501126)
IL-27 Goat anti-Human, Polyclonal, R&D Systems™	RnD systems	RnD systems Cat# Af2526 Goat anti-human polyclonal antibody
InVivoMAb mouse IgG1 isotype control	BioXCell	BioXCell Cat#BE0083 Clone MOPC-21 RRID: AB_1107784
InVivoMAb anti-human PD-1 (CD279)	BioXCell	BioXCell Cat#BE0188 Clone J116 RRID AB_10950318
InVivoMAb anti-human PD-L1 (B7-H1)	BioXCell	BioXCell Cat#BE0285 Clone 29E.2A3 RRID AB_2687808
InVivoMAb mouse IgG2b isotype control	BioXCell	BioXCell Cat#BE0086 Clone MPC-11 RRID AB_1107791
InVivoMAb anti-human/monkey CD28	BioXCell	BioXCell Cat#BE0291 Clone CD28.2 RRID AB_2687814
InVivoMAb anti-human CD3	BioXCell	BioXCell Cat#BE0291Clone OKT-3 RRID AB_1107632
APC-Cy™7 Mouse Anti-Human CD45	BD	BD Biosciences Cat# 557833, RRID:AB_396891Clone 2D1
Alexa Fluor® 700 anti-human CD3 antibody, BioLegend	Biolegend	BioLegend Cat# 300424, RRID:AB_493741 Clone UCHT1
Brilliant Violet 421™ anti-human CD8 antibody, BioLegend	Biolegend	BioLegend Cat# 344748, RRID:AB_2629584 Clone SK1
Brilliant Violet 711™ anti-human CD4 antibody, BioLegend	Biolegend	BioLegend Cat# 317440, RRID:AB_2562912 Clone OKT4
APC anti-human CD103 (Integrin alphaE) antibody	Biolegend	BioLegend Cat# 350216, RRID:AB_2563907 Clone BER-ACT8
Brilliant Violet 605™ anti-human CD39 antibody	Biolegend	BioLegend Cat# 328236, RRID:AB_2750430 Clone A1
PE anti-human CD279 (PD-1) antibody	Biolegend	BioLegend Cat# 329905, RRID:AB_940481 Clone EH12.2H7
PE/Dazzle™ 594 anti-human CD366 (Tim-3) antibody	Biolegend	BioLegend Cat# 345034, RRID:AB_2565886 Clone F38-2E2
BV650 anti-CD223 LAG3	Biolegend	BioLegend Cat# 369315, RRID:AB_2632950 Clone 11C3C65
FITC Anti-human CD49b	Biolegend	BioLegend Cat# 359306, RRID:AB_2562531 Clone P1E6-C5
Brilliant Violet 605™ anti-human CD25 antibody	Biolegend	BioLegend Cat# 302631, RRID:AB_11123913 Clone BC96
PerCP/Cyanine5.5 anti-human CD127 (IL-7Ralpha) antibody	Biolegend	BioLegend Cat# 351321, RRID:AB_10900253 Clone A019D5
Mouse Anti-Human Epcam/trop-1 Monoclonal antibody, Fluorescein Conjugated	RnD systems	R and D Systems Cat# FAB9601 F, RRID:AB_2246506 Clone 158206
FITC anti-human CD31 antibody	Biolegend	BioLegend Cat# 303104, RRID:AB_314330 Clone WM59
Human Fibroblast Activation Protein alpha /FAP PE-conjugated Antibody	RnD systems	RnD systems Cat# FAB3715P Clone # 427819
CD90 Antibody, anti-human, VioBlue®, REAfinity™	Miltenyi	Miltenyi Biotec Cat# 130–114-866, RRID:AB_2726816 Clone [REA897]
FITC anti-human HLA-A,B,C antibody	Biolegend	BioLegend Cat# 311404, RRID:AB_314873 Clone W6/32
Brilliant Violet 650™ anti-human HLA-DR antibody	Biolegend	BioLegend Cat# 307649, RRID:AB_2562544 Clone L243
CD273 (B7-DC) Monoclonal Antibody (MIH18), PE,	Invitrogen	Thermo Fisher Scientific Cat# 12–5888-42, RRID:AB_10853342 Clone MIH18
CD274 (PD-L1, B7-H1) Monoclonal Antibody (MIH1), APC, eBioscience™	eBioscience	Thermo Fisher Scientific Cat# 17–5983-42, RRID:AB_10597586 Clone MIH1
PerCP/Cyanine5.5 anti-human CD73 (Ecto-5’-nucleotidase) antibody	Biolegend	BioLegend Cat# 344013, RRID:AB_2561756 Clone AD2
PE anti-human IFN-gamma antibody	Biolegend	BioLegend Cat# 502509, RRID:AB_315234 Clone 4S.B3
PerCP/Cyanine5.5 anti-human IL-10 antibody	Biolegend	BioLegend Cat# 501417, RRID:AB_2561285 Clone JES3-9D7
Alexa Fluor(R) 488 anti-human TNF-alpha antibody	Biolegend	BioLegend Cat# 502915, RRID:AB_493121 clone 2A5
Pacific Blue™ anti-human/mouse Granzyme B antibody	Biolegend	BioLegend Cat# 515408, RRID:AB_2562196 Clone GB11
BV605 Mouse IgG1, κ Isotype Control antibody	BD	BD Biosciences Cat# 562652, RRID:AB_2714005 Clone X40
Ki-67 Monoclonal Antibody (20Raj1), eBioscience™	eBioscience	Thermo Fisher Scientific Cat# 14–5699-82, RRID:AB_2016711 Clone 20Raj1
PE anti-human FOXP3 antibody	Biolegend	BioLegend Cat# 320108, RRID:AB_492986 Clone 206D
Brilliant Violet 711™ anti-human IL-2 antibody	Biolegend	BioLegend Cat# 500345, RRID:AB_2616638 Clone MQ1-17H12
PerCP/Cyanine5.5 Rat IgG1, κ Isotype Ctrl Antibody	Biolegend	Biolegend Cat# 400426 Clone RTK 7021
Alexa Fluor® 700 anti-human IL-2 antibody	Biolegend	BioLegend Cat# 500320, RRID:AB_528929 Clone MQ1-17H12
Alexa Fluor® 488 Mouse IgG1, κ Isotype Ctrl (FC) Antibody	Biolegend	Biolegend Cat# 400129 Clone MOPC-21
APC Rat IgG2a, κ Isotype Ctrl Antibody	Biolegend	BioLegend Cat# 400512, RRID:AB_2814702 Clone [RTK2758]
PE Mouse IgG1, κ Isotype Ctrl Antibody	Biolegend	BioLegend Cat# 400112, RRID:AB_2847829Clone MOPC-21
Human TruStain FcX™ (Fc Receptor Blocking Solution) antibody	Biolegend	BioLegend Cat# 422302, RRID:AB_2818986
Brilliant Violet 605™ anti-human CD326 (Ep-CAM) antibody	Biolegend	BioLegend Cat# 324224, RRID:AB_2562518 Clone 9C4
APC/Cyanine7 anti-human IL-17A antibody	Biolegend	BioLegend Cat# 512319, RRID:AB_10612577 Clone BL168
Fixation Buffer	Biolegend	Biolegend Cat # 420801
Zombie UV Viability Kit	Biolegend	Biolegend Cat # 423108
ELISA MAX deluxe Human IFN-gamma ELISA	Biolegend	Biolegend Cat # 430104
ELISA MAX deluxe Human IL-10 ELISA	Biolegend	Biolegend Cat # 430604
ELISA MAX deluxe Human IL-6 ELISA	Biolegend	Biolegend Cat # 430504
Cyto-last buffer	Biolegend	Biolegend Cat # 422501
One comp eBeads	Thermo Fisher	Thermor Fisher Cat # 01–1111-42
Human IL-27 DuoSet ELISA	RnD systems	RnD systems Cat # DY2526-05
Cytofix/cytoperm Fixation/Permeabilization Solution Kit	BD	BD Cat # 554714
HBSS	Gibco	Gibco Cat # 14170–088
PBS – cal – mg	Gibco	Gibco Cat # 14190–094
RPMI	Gibco	Gibco Cat # 31870–025
DMEM	Gibco	Gibco Cat # 21969–035
CD4 microbeads human	Miltenyi	Miltenyi Cat # 130–045-101
CD8 microbeads human	Miltenyi	MiltenyiCat # 130–045-201
IL-2 recombinant protein	Gibco	Gibco Cat # PHC0026
FCS	Gibco	Gibco Cat # 10500–064
Pen-Strep	Gibco	Gibco Cat # 15140–122
L-glutamine	Gibco	Gibco Cat # 25030–024
eBioscience™ Cell Stimulation Cocktail (500X)	Thermo Fisher	Thermo Fisher Cat # 00–4970-93
Collagenase IV	Worthington	Merck Cat # C5138
Collagenase I	Sigma	Merck Cat # SCR 103
DNAse	Sigma	Merck Cat # DN25
Hyaluronidase	Stem Cell technologies	Stem cell technologies Cat # 7461
Lymphoprep	Stem Cell technologies	Stem cell Catalog # 07801
RBC lysis buffer	Sigma	Merck Cat# 11814389001
Falcon™ Cell Strainers 100 uM	Thermo Fisher	Falcon Cat # 10282631
Falcon™ Cell Strainers 70 uM	Thermo Fisher	Falcon Cat # 10788201

**Table:** 

**Antibody**	**Concentration**	**Supplier**	**Reference**
FAP	1:150–250	R and D	AF3715
CD3	1:200	Abcam	Ab11089
CD8	1:100	Leica Biosystems	NCL-L-CD8-4B11
CD39	1:100	Biolegend	328202
CD103	1:250	Abcam	ab129202
Pan-CK	1:1500	Thermofisher	53–9003-80
Tyramide A488	1:200	Thermofisher	B40912
Qdot 605	1:200	Thermofisher	Q10103MP
Qdot 585	1:100	Thermofisher	Q10113MP
Qdot 565	3:100	Thermofisher	Q10133MP
Qdot 705	1:200	Thermofisher	Q10163MP
Qred	1:750	Thermofisher	Q10363
Secondary α-Sheep biotinylated	Pre-diluted	R and D	CTS019
Secondary αMouse biotinylated	1:200	Abcam	ab6822
Secondary α Rabbit biotinylated	1:200	Abcam	ab7055
Secondary αRat biotinylated	1:500	Vector	BA-9401
Mouse Fab	1:50	Jackson	715–007-003
Rabbit Fab	1:50	Jackson	711–007-003

## Supplementary Material

Supplemental Material
