# Supplementary material for: T cells drive negative feedback mechanisms in cancer associated fibroblasts, promoting expression of co-inhibitory ligands, CD73 and IL-27 in non-small cell lung cancer
Source: Oncoimmunology. 2021 Jul 8;10(1):1940675. doi: 10.1080/2162402X.2021.1940675 (PMC8274440; doi:10.1080/2162402X.2021.1940675)
Supplement: Supplemental Material [file KONI_A_1940675_SM6402.zip › Supplementary Information.docx]

**Supplementary Information**

**T cells drive negative feedback mechanisms in Cancer Associated Fibroblasts, promoting expression of co-inhibitory ligands, CD73 and IL-27 in non-small cell lung cancer.**

Richard A O’Connor, Vishwani Chauhan, Layla Mathieson, Helen Titmarsh, Lilian Koppensteiner, Irene Young, Giulia Tagliavini, David A Dorward, Sandrine Prost, Kevin Dhaliwal, William A Wallace, Ahsan R Akram.

**Supplementary Figures 1-8.**


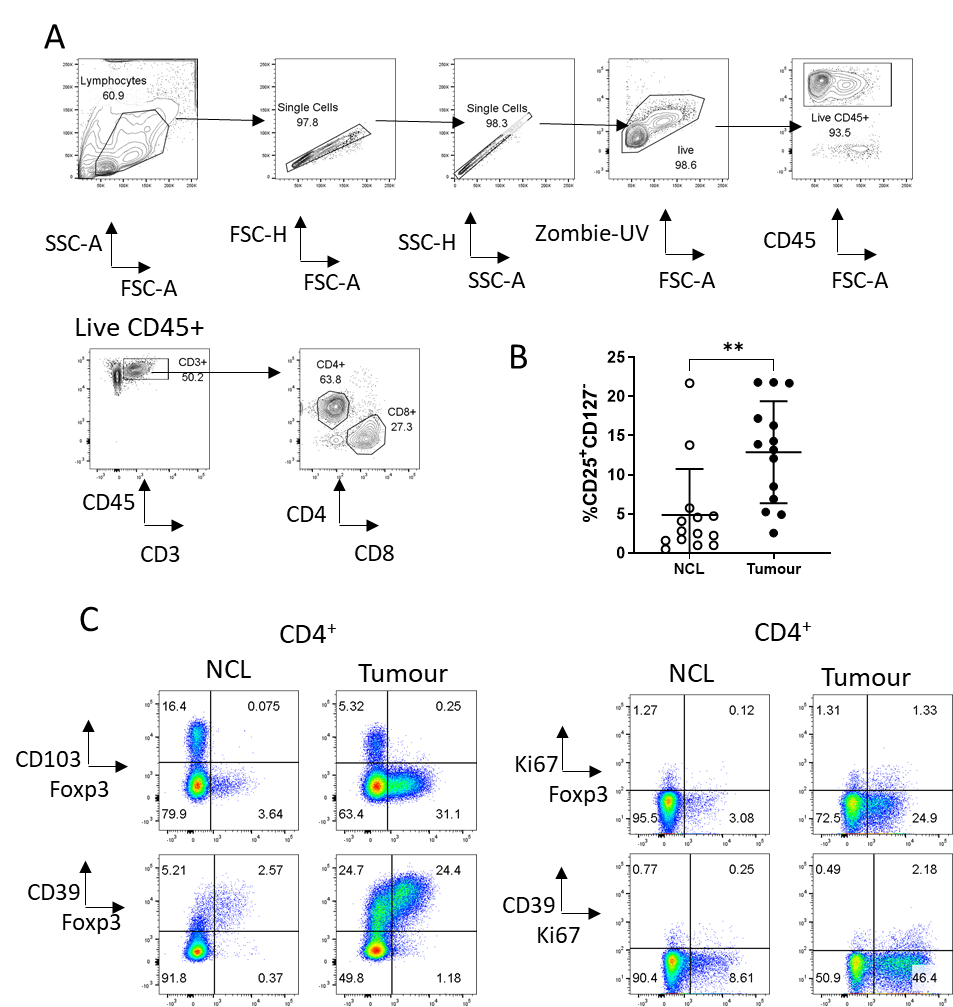


**Sup Fig. 1. CD39^+^ Tregs are enriched within NSCLC tumours.** A) Gating strategy to identify T cells in tissue digests. B) Frequency of CD25^+^ CD127^-^ Tregs in non-cancerous lung tissue (NCL) and paired NSCLC tumour samples (n=14) (***P*=0.0059) two tailed paired T test was used to analyse data. C) Phenotype of CD4^+^ T cells from non-cancerous lung tissue and paired NSCLC tumour sample showing expression of Foxp3 with CD103, CD39 and Ki67 and expression of CD39 and Ki67.


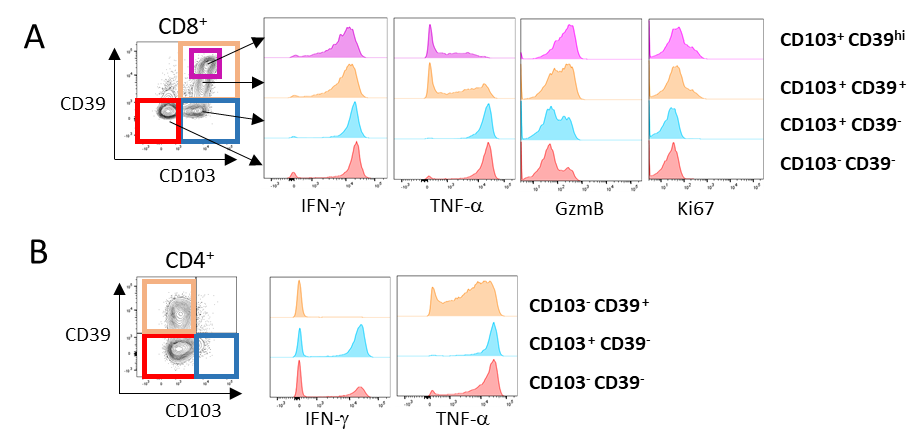


**Sup Fig. 2. Cytokine production in tumour infiltrating T cells.** Single cell suspensions from NSCLC tumour digest were stimulated with PMA / Ionomycin in the presence of Brefeldin A for four hours prior to cell surface staining, fixation and staining for intracellular cytokines. A) Tumour infiltrating CD8^+^ T cells were gated as shown in the left hand panel defining four populations of interest: CD103^-^CD39^-^, CD103^+^CD39^-^, CD103^+^CD39 ^+^ and CD103^+^CD39^hi^. Expression profiles of IFN-g, TNF-a, Granzyme B and Ki67 within these populations are shown. B) Tumour infiltrating CD4^+^T cells were gated into three subpopulations as shown in the left hand panel CD103^-^CD39^-^, CD103^-^CD39^+^ and CD103^+^CD39^-^. Expression profiles of IFN-ɣ and TNF-α within these populations are shown.


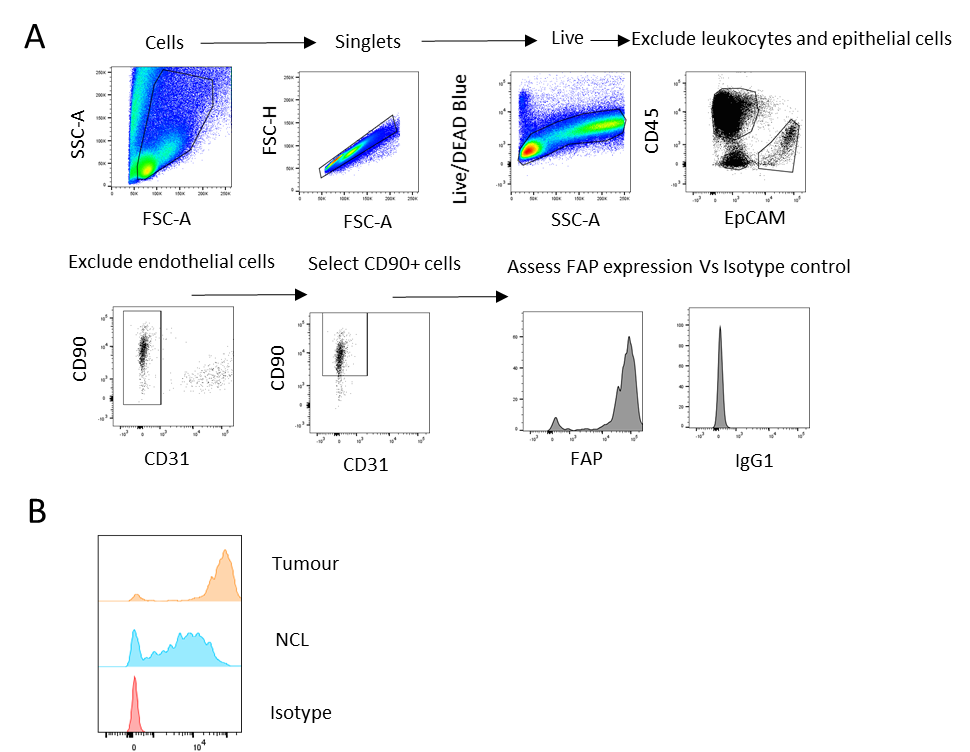


**Sup Fig. 3. Gating strategy for identification of cancer associated fibroblasts.** A) Gating strategy for fibroblasts; debris was excluded then singlets were selected using FSC-A versus FSC-H, live cells were selected as Zombie-UV- as shown, CD45^+^ and Epcam^+^ cells were excluded, CD31^+^ endothelial cells were excluded and CD90^+^ cells selected as fibroblasts. The profile of FAP staining or isotype control staining on CD90^+^ cells is shown. B) Representative staining for FAP-expression on live CD45^-^EpCAM^-^CD31^-^CD90^+^ cells from NSCLC tumour or paired non-cancerous lung (NCL) sample is shown.


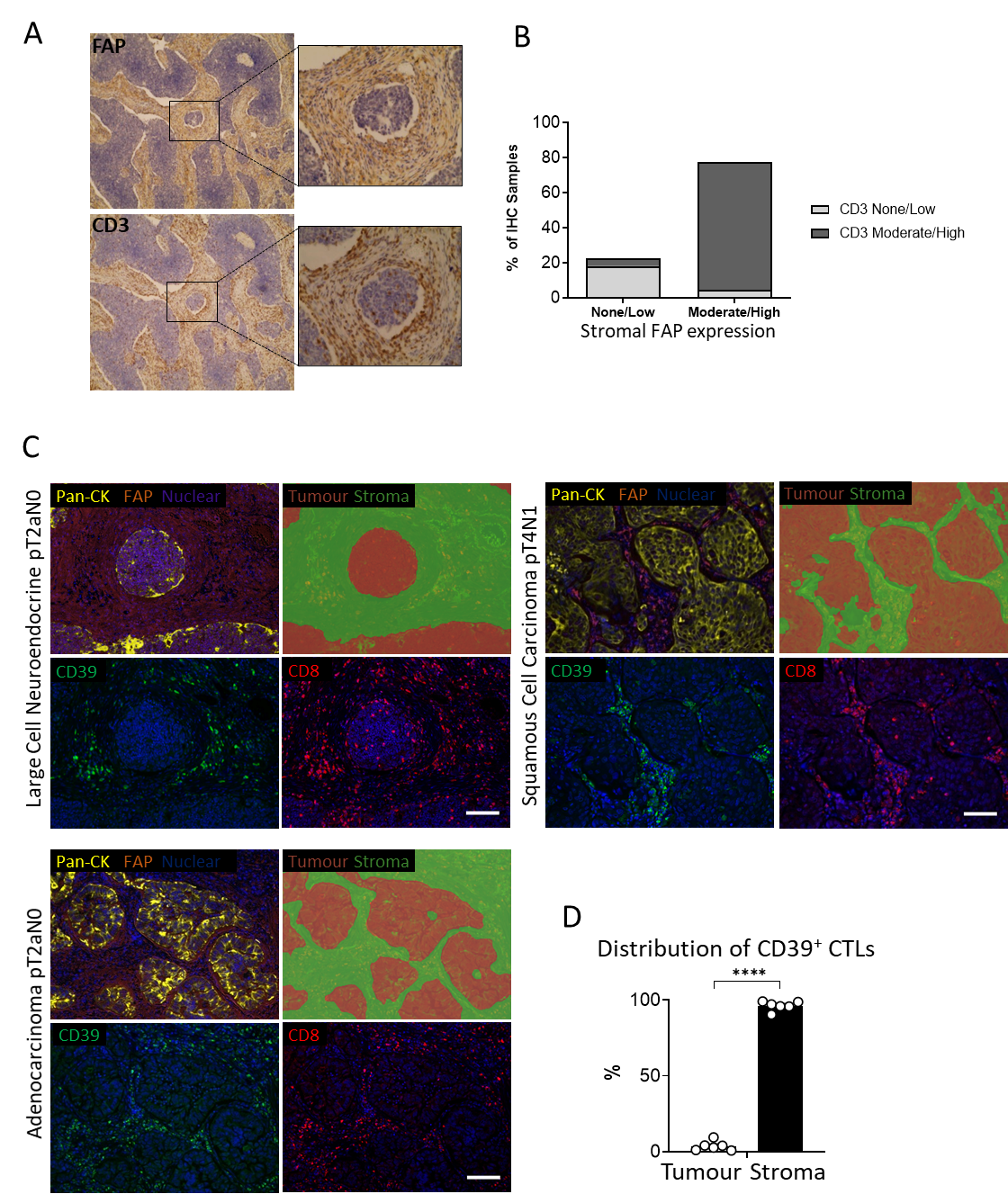


**Sup Fig. 4. Localisation of CD39^+^ CTLs within the FAP rich stroma in NSCLC.** A) FAP and CD3 staining of NSCLC tumour (pT2aN0 large cell neuroendocrine) with enlargement of marked area showing the majority of CD3^+^ T cells are found within the stroma. B) NSCLC tumours from 22 patients were stained for FAP and CD3 on consecutive slices and scored for intensity within the stroma; 0 (none), 1 (low), 2 (moderate) and 3 (high). T cell infiltration was seen at low levels in tumours with FAP-low stroma and at high levels in tumours with FAP-high stroma. C) Immunohistochemistry showing distribution of CD39^+^ cells and CD8^+^ T cells in tumour and stroma regions. Representative images shown, scale bar 100 µm. d) Proportion of CD39^+^CD8^+^ T cells located in tumour of stroma of six FAP^hi^ NSCLC tumours, two tailed paired t-test, ****=p<0.001.

**Sup Fig. 5. Pre-exposure of CAF to IFN-γ and TNF-α does not enhance their capacity to induce co-inhibitory receptors on activated T cells.** CAFs were cultured in the presence or absence of IFN-γ and TNF-α for 48 hrs prior to harvesting. After being re-plated and allowed to adhere overnight PBMC were added and stimulated with anti-CD3 / Anti-CD28. The proportion of CD8+ (A) and CD4+ (B) T cells expressing the indicated co-inhibitory receptors after 48 hrs stimulation in the presence of untreated (open bars) / or pre-treated (grey bars) CAFs is shown. Graphs show the mean and standard deviation of triplicate wells.


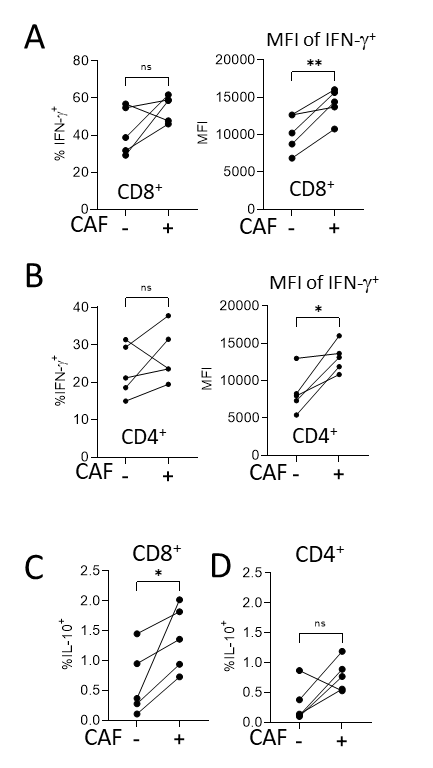


**Sup Fig. 6. Production of IFN-g and IL-10 by T cells stimulated in the presence or absence of CAF.** A) % of cells positive for IFN-g and the MFI of IFN-g staining in IFN-g ^+^ cells in CD8^+^ T cells stimulated in the presence or absence of CAFs. B) % of cells positive for IFN-g and the MFI of IFN-g staining in IFN-g^+^ cells in CD4^+^ T cells stimulated in the presence or absence of CAFs. C) % of CD8^+^ T cells positive for IL-10 stimulated in the presence or absence of CAFs. D) % of CD4^+^ T cells positive for IL-10 stimulated in the presence or absence of CAFs. Analyses by two tailed paired t-test, ns=not significant, *=p<0.05, **=p<0.01.

**Sup Fig. 7. IL-27 does not promote CD39 expression in T cells stimulated in the presence of CAFs.** Expression of CD39 in CD8+ (A) and CD4+ (B) T cells amongst PBMC cultured in the presence (Stim) or absence (Unstim) of anti-CD3 / anti-CD28 antibodies with addition of 10 ng / ml recombinant IL-27 as indicated. Expression of CD39 among CD8+ (C) and CD4+ (D) T cells among PBMC stimulated with anti-CD3 / anti-CD28 alone or in the presence of CAF, with neutralising anti-IL27 or isotype matched control antibody (10 μg/ml), or of additional rIL-27 (10 ng/ml). E) production of IL-10 (Left) and IL-17 (Right) by PBMC after 48 hrs culture in the presence or absence of 10 ng / ml rIL-27 with or without anti-CD3 / anti-CD28 (STIM) either alone or in co-culture with CAF in the presence of rIL-27 or anti-IL-27 (10 μg/ml). Mean and standard deviation of triplicate cultures are shown.

A

B

C

D

E

**Sup Fig. 8. IL-27 does not alter expression of PD-L1, PD-L2, CD80, CD39 or CD73 by CAF.** Mean fluorescence intensity (MFI) of PD-L1 (A), PD-L2 (B), CD80 (C), CD39 (D) and CD73 (E) on CAF cultured for 48 hrs in increasing concentrations of recombinant IL-27 (as indicated). Results shown are from three CAF lines and are representative of two independent experiments.
